# Uncovering Sternoclavicular Arthritis, Suspected Pseudogout, in a Fever of Unknown Origin by Whole-Body MRI

**DOI:** 10.3390/diagnostics15162032

**Published:** 2025-08-13

**Authors:** Maho Hayashi, Koji Hayashi, Mamiko Sato, Toshiko Iwasaki, Yasutaka Kobayashi

**Affiliations:** 1 Department of Internal Medicine, Fukui General Hospital, 55-16-1 Egami-cho, Fukui 910-8561, Japan; 2 Department of Rehabilitation Medicine, Fukui General Hospital, 55-16-1 Egami-cho, Fukui 910-8561, Japan; satomoko@f-gh.jp; 3 Graduate School of Health Science, Fukui Health Science University, 55-13-1 Egami-cho, Fukui 910-3190, Japan; yasutaka_k@fukui-hsu.ac.jp; 4 Department of Radiology, Fukui General Hospital, 55-16-1 Egami-cho, Fukui 910-8561, Japan; a.r.w.tiwasaki@gmail.com

**Keywords:** pseudogout, sternoclavicular joint, arthritis, fever of unknown origin, MRI

## Abstract

An 89-year-old male developed a persistent high fever (around 39 °C) approximately two weeks following endoscopic reduction of sigmoid volvulus. He had no history of hypercalcemia but was using diuretics and proton pump inhibitors. Renal and thyroid status were normal. He was largely bedridden and asymptomatic except for fever. Laboratory tests demonstrated elevated C-reactive protein (4.75 mg/dL), but some tumor markers (including CEA, CA19-9, and CA125), anti-nuclear antibodies, MPO-ANCA, PR3-ANCA, β-D-glucan, and interferon-gamma release assay were all negative. Urinalysis was unremarkable. Blood cultures obtained from two sets were negative. Chest–abdomen–pelvis contrast-enhanced computed tomography (CT), and echocardiography did not reveal any evident neoplastic lesions or focal sites of infection. Despite various antibiotic therapies, the patient’s spike fever persisted for nearly one month, leading to a diagnosis of fever of unknown origin (FUO). The patient experienced partial symptomatic relief with corticosteroid therapy, though mild fever continued. Two months after the volvulus onset, diffusion-weighted whole-body imaging with background body signal suppression (DWIBS) was performed, revealing hyperintensities at the right sternoclavicular joint, leading to a diagnosis of sternoclavicular arthritis. Neck CT revealed calcification in this joint. Despite difficulty in joint fluid analysis, low infection risk and the patient’s prolonged bedridden state and advanced age led to suspicion of pseudogout. Nonsteroidal anti-inflammatory drugs relieved fever and normalized inflammatory markers. DWIBS may be a valuable tool for detecting potential focus sites in FUO.

**Figure 1:**
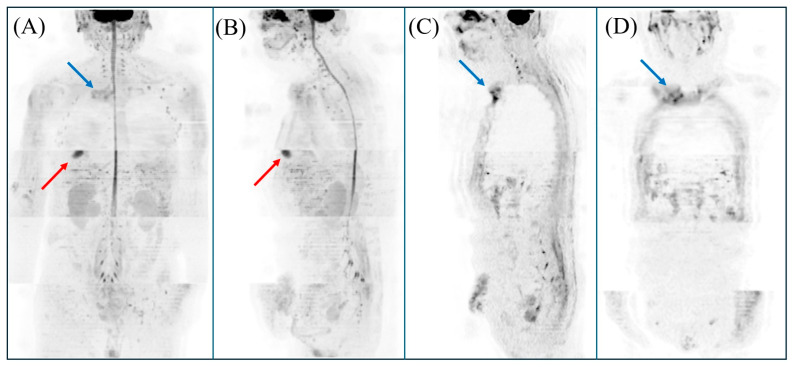
The results of magnetic resonance imaging (MRI) on diffusion-weighted whole-body imaging with background body signal suppression (DWIBS) are shown. For our DWIBS examination, the specific imaging protocol included a b-value of 1000 s/mm^2^, an echo time (TE) of 90.2 ms, a repetition time (TR) of 7500 ms, and a slice thickness of 5 mm. The total scan duration for this sequence was approximately 32 min and 56 s. (**A**,**B**) Three-dimensional rendering of DWIBS data demonstrating hyperintensities in the right sternoclavicular joint (blue arrow) and the right anterior chest subcutaneous (red arrow). (**A**) Coronal section; (**B**) sagittal section. (**C**,**D**) Diffusion-weighted hyperintensity lesion was noted in the right sternoclavicular joint (blue arrow). Based on these findings, the patient was diagnosed with both sternoclavicular arthritis and a sebaceous cyst; however, the presence of sternoclavicular arthritis is more likely to be related to the fever. DWIBS is an advanced MRI technique, first developed by Takahara et al. in 2004 [1], that provides valuable functional information applicable across a range of clinical settings [2,3]. It is based on diffusion-weighted imaging (DWI), which monitors the random movement of water molecules within tissues (Brownian motion) [2,4,5]. The method uses a short τ inversion recovery (STIR) echo-planar imaging (EPI) sequence with free breathing, allowing for image acquisition without the need for respiratory restrictions [2,3,4,5]. This approach employs multiple signal averages, fat suppression, and heavy diffusion weighting to diminish background signals from normal organs. This enhances contrast between healthy and abnormal tissues [2,4,5]. Such suppression highlights abnormal areas, such as tumors, acute inflammatory regions, or abscesses. These appear as high-intensity signals against the subdued background, facilitating easier detection [2,3,4,5,6]. It can generate multiple thin-slice diffusion-weighted images and compile them into a three-dimensional, PET-like visualization [3]. Recent consensus guidelines for FUO emphasize that, if available, chest, abdominal, and pelvic CT should be preferred over chest plain radiography or abdominal ultrasonography as the imaging component for the minimum diagnostic criteria for FUO [7,8]. Furthermore, 18FDG-PET/CT is considered an important early diagnostic test after a patient fulfills the FUO criteria with minimal diagnostic tests, particularly in the absence of potential diagnostic clues. DWIBS, with its many advantages over other modalities—including the fact that it is non-invasive, relatively inexpensive, requires no contrast agents, does not expose patients to radiation, and enables comprehensive whole-body evaluation [2,3,4]—may hold promise as a valuable diagnostic tool for FUO, complementing, or potentially preceding, other advanced imaging techniques [9]. Reports evaluating inflammation using DWIBS include aortitis (Takayasu arteritis and stent-associated aortitis), acute cholecystitis, bacterial nephritis, myocardial abscess, and sacroiliitis [2,3,4,5,10,11,12].

**Figure 2:**
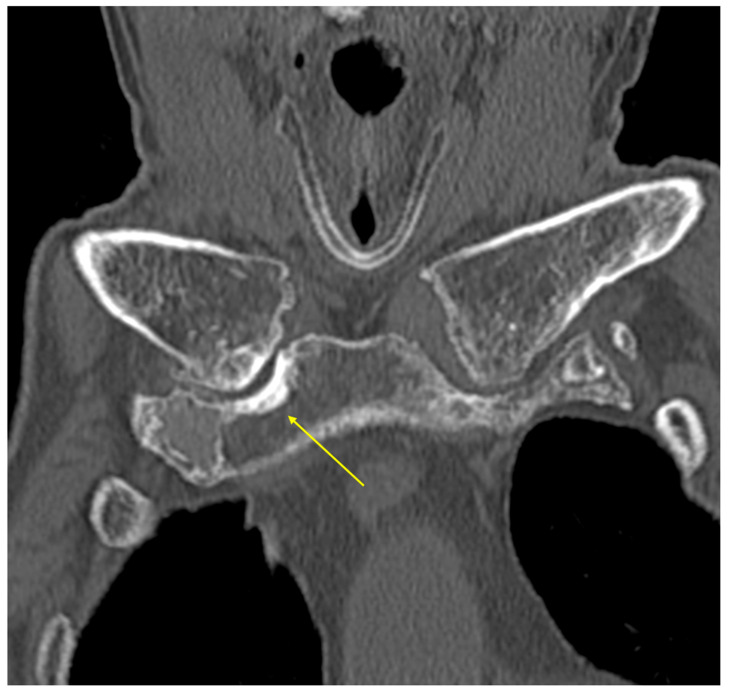
Neck CT three days after taking DWIBS reveals calcification in the right sternoclavicular joint (arrow). Inflammation of the sternoclavicular joint can be caused by infections, rheumatoid arthritis, SAPHO syndrome, or crystal arthritis [13]. In our case, arthritis was limited to the right sternoclavicular joint, and no symptoms (including joint deformity or swelling) were noted in the four extremities. No skin lesions or other joints were affected. In addition, autoimmune antibodies, including anti-nuclear antibodies, MPO-ANCA and PR3-ANCA, were negative. Therefore, autoimmune arthritis (including rheumatoid arthritis or SAPHO syndrome) was less likely. Additionally, the condition was refractory to a wide variety of antibiotics, and blood cultures were negative, making infectious causes unlikely. Therefore, crystal arthritis is considered more probable than other etiologies. Although the precise etiology of sternoclavicular remains unknown because fluid sampling from the sternoclavicular joint was not performed, the patient’s advanced age, prolonged bedridden state, good response to NSAIDs, antidiuretic use, and the presence of joint calcification support a diagnosis of pseudogout [14,15].

## Data Availability

The data presented in this study are available on request from the corresponding author. Due to patient privacy and ethical considerations, the data are not publicly accessible.

## Abbreviations

The following abbreviations are used in this manuscript:

CT	Computed tomography
MRI	Magnetic resonance imaging
DWIBS	Diffusion-weighted whole-body imaging with background body signal suppression
FUO	Fever of unknown origin
